# Genome report: chromosome-level draft assemblies of the snow leopard, African leopard, and tiger (*Panthera uncia*, *Panthera pardus pardus*, and *Panthera tigris*)

**DOI:** 10.1093/g3journal/jkac277

**Published:** 2022-10-17

**Authors:** Ellie E Armstrong, Michael G Campana, Katherine A Solari, Simon R Morgan, Oliver A Ryder, Vincent N Naude, Gustaf Samelius, Koustubh Sharma, Elizabeth A Hadly, Dmitri A Petrov

**Affiliations:** Department of Biology, Stanford University, Stanford, CA 94305, USA; Department of Biology, Washington State University, Pullman, WA 99164, USA; Center for Conservation Genomics, Smithsonian’s National Zoological Park and Conservation Biology Institute, Washington, DC 20008, USA; Department of Biology, Stanford University, Stanford, CA 94305, USA; Department of Biology, Stanford University, Stanford, CA 94305, USA; Wildlife ACT Fund Trust, Cape Town 8001, South Africa; San Diego Zoo Wildlife Alliance, Beckman Center for Conservation Research, San Diego, CA 92027, USA; Department of Conservation Ecology and Entomology, University of Stellenbosch, Stellenbosch, 7602, South Africa; School of Animal, Plant and Environmental Sciences, University of the Witwatersrand, Johannesburg 2000, South Africa; Snow Leopard Trust, Seattle, WA 98103, USA; Snow Leopard Trust, Seattle, WA 98103, USA; Nature Conservation Foundation, Mysore 570 017, India; Department of Biology, Stanford University, Stanford, CA 94305, USA; Department of Biology, Stanford University, Stanford, CA 94305, USA

**Keywords:** *Panthera uncia*, *Panthera pardus pardus*, *Panthera tigris*, big cats, genome assembly

## Abstract

The big cats (genus *Panthera*) represent some of the most popular and charismatic species on the planet. Although some reference genomes are available for this clade, few are at the chromosome level, inhibiting high-resolution genomic studies. We assembled genomes from 3 members of the genus, the tiger (*Panthera tigris*), the snow leopard (*Panthera uncia*), and the African leopard (*Panthera pardus pardus*), at chromosome or near-chromosome level. We used a combination of short- and long-read technologies, as well as proximity ligation data from Hi-C technology, to achieve high continuity and contiguity for each individual. We hope that these genomes will aid in further evolutionary and conservation research of this iconic group of mammals.

## Introduction

The genus *Panthera* comprises 5 extant species of Felidae and forms the majority of the group colloquially termed “big cats”. The clade is thought to have diverged approximately 5 million years ago (Johnson *et al.* 2006; Davis *et al.* 2010) and consists of some of the most widespread and successful carnivores on the planet. However, over the last 100 years, *Panthera* species have suffered widespread and severe declines, primarily due to anthropogenic causes (Sunquist and Sunquist 2002). As large, top terrestrial predators, members of the *Panthera* clade have naturally low abundances and slower intrinsic rates of population growth compared to prey species, forcing many populations into “threatened” or “endangered” listings by the International Union for Conservation of Nature (IUCN) and Convention on International Trade in Endangered Species (CITES), with severe ecological implications globally.

Due to their morphological similarities (not including their considerable variation in coat color and pattern), and the unresolved paleontological history of the big cats, the genus *Panthera* has undergone substantial taxonomic rearrangements over the last century. Initially (1816–1916), the clade contained all spotted cats, irrespective of size or other morphological differences (Allen 1902). Later (1916), the clade was redefined to include only the jaguar (*Panthera onca*), the lion (*Panthera leo*), the leopard (*Panthera pardus*), and the tiger (*Panthera tigris*) (Pocock 1916). At various times, arguments for the inclusion of the clouded leopard (*Neofelis nebulosa*) and the snow leopard (*Panthera uncia*) surfaced, but availability of more genetic data supported the inclusion of only the snow leopard, defining the clade that we know today (jaguar, lion, leopard, tiger, snow leopard) (Johnson *et al.* 2006; Davis *et al.* 2010; Kitchener *et al.* 2017). Despite the refinement of the general tree topology, genomic data have been used to show that potential postspeciation hybridization and/or incomplete lineage sorting (ILS) appear to have occurred frequently during the evolution of the genus (Li *et al.* 2016; Figueiró *et al.* 2017; Li *et al.* 2019). Very recent demographic events or complex demographic histories require haplotype-level information to resolve, such as is provided by contiguous, chromosome-level assemblies.

To date, several draft genome assemblies have been published for members of *Panthera*, but few are at the chromosome level. Of those that are at the chromosome level, many contain unresolved regions, reflected by low contig statistics (e.g. contig N50, contig L50). Draft genome assemblies have previously been published for the jaguar (Figueiró *et al.* 2017), lion (Armstrong *et al.* 2020), tiger (Cho *et al.* 2013; Armstrong *et al.* 2019; Khan *et al.* 2021), snow leopard (Cho *et al.* 2013), and leopard (Kim *et al.* 2016). However, only assemblies for the lion (GCA_018350215.1, Armstrong *et al.* 2020) and the tiger (GCA_018350195.2) are highly contiguous and at the chromosome level. High-quality genomes have been shown to aid in the resolution of phylogenetic history, recently and notably identifying that giraffes (*Giraffa* spp.) are composed of several distinct lineages (Coimbra *et al.* 2021) and that the forest (*Loxodonta cyclotis*) and savannah elephant (*Loxodonta africana*) have been separated for 2.5 million years (Rohland *et al.* 2010). Thus, improved high-quality assemblies will help to unravel the complex divergence history of *Panthera* and will additionally be useful for conservation applications, such as population genetics and forensic monitoring of wildlife products.

The tiger (*P. tigris*) comprises 6 extant subspecies, of which one (South China tiger, *Panthera tigris amoyensis*) is functionally extinct in the wild and only exists in captivity (Tilson *et al.* 1997). Currently, draft reference genomes exist for the Bengal (*Panthera tigris tigris*) (Anuradha Reddy *et al.* 2018; Khan *et al.* 2021), Amur (*Panthera tigris altaica*) (Cho *et al.* 2013), and Malayan (*Panthera tigris jacksoni*) (Armstrong *et al.* 2019), but not the Sumatran (*Panthera tigris sumatrae*) or Indochinese (*Panthera tigris corbetti*) tiger subspecies. Snow leopards (*P. uncia*), found in the high mountains of south-central Asia, are experiencing increasing anthropogenic threats and it is estimated that only about 3,500–7,500 of these cats remain in the wild (McCarthy *et al.* 2003; Jackson *et al.* 2010; Macdonald and Loveridge 2010; Zakharenka *et al.* 2016). At present, 1 fragmented genome assembly exists for the snow leopard, courtesy of DNA Zoo (dnazoo.org). The leopard (*P. pardus*), the most widely distributed big cat, consists of 9 extant subspecies (Kitchener *et al.* 2017). It is thought that African leopards (*Panthera pardus pardus*) gave rise to 8 Middle-Eastern (Arabian, *Panthera pardus nimr*; Persian, *Panthera pardus saxicolor*) and Asian (Indian, *Panthera pardus fusca*; Sri Lankan, *Panthera pardus kotiya*; Indochinese, *Panthera pardus delacouri*; North-Chinese, *Panthera pardus japonensis*; Amur, *Panthera pardus orientalis*; and Javan, *Panthera pardus melas*) subspecies around 500–600 thousand years ago (Paijmans *et al.* 2021). There are remarkably few genomic resources for leopards, with only a single fragmented *de novo* assembly having been published to date (Kim *et al.* 2016).

Here, we add to the chromosome-level genome repertoire of *Panthera* with novel chromosome-level assemblies for a tiger of generic (mixed) ancestry, a snow leopard, and an African leopard. These resources will help guide further research into the evolutionary history and conservation of these big cats.

## Materials and methods

### Tiger sequencing

The tiger sample used for whole-genome assembly was provided by In-Sync Exotics (Wylie, TX, USA) from a male cub named “Kylo Ren” with generic ancestry (Table 1). An EDTA whole-blood sample was taken during a routine veterinary check, subsequently packed on ice, and shipped to the Petrov and Hadly labs at Stanford University (Stanford, CA, USA). An aliquot of this sample was then shipped on ice to Hudson Alpha (Huntsville, AL, USA) for 10× Genomics Chromium library preparation. This sample was extracted using the Qiagen MagAttract kit (Cat # 67563). A Chromium library was prepared using standard instructions, shipped to Admera Health (Plainsfield, NJ, USA) and bidirectionally sequenced using 150-bp reads on a lane of HiSeq X Ten (Illumina, Inc., San Diego, CA, USA).

**Table 1.** Sample information for individuals used in sequencing and de novo assembly.

Species	Sample origin	Sample ID	Sex	Name	Ancestry	Identification #
*Panthera tigris*	In-Sync Exotics (Wylie, Texas)	GenTig	Male	Kylo Ren	Mixed, Sanctuary	NA
*Panthera uncia*	San Diego Frozen Zoo (San Diego, CA)	Puncia	Female	Amy	Captive, AZA Zoo	SB1802
*Panthera pardus pardus*	Maryland Zoo (Baltimore, Maryland)	Ppardus1	Male	Hobbes	South Africa/Allsday District, Northern Province	GAN: MIG12-29918358
*Panthera pardus pardus*	Maryland Zoo (Baltimore, Maryland)	Ppardus2	Female	Amari	South Africa/Thornybush, E. Transvaal	GAN: MIG12-29918144

Material not used for 10× Genomics preparation was then used to construct a Hi-C library at Stanford University. Approximately 2 ml of whole blood was used as input in the Dovetail Hi-C kit (Cat # 21004) and a library was prepared according to the provided instructions. The finished library was then sent to Admera Health and bidirectionally sequenced using 150-bp reads on a HiSeq X Ten lane.

### Snow leopard sequencing

An aliquot of a snow leopard cell line (ISIS # 500107, Table 1) was obtained from the San Diego Frozen Zoo. The sample was sent to the Stanford Functional Genomics Facility (SFGF) for high molecular weight (HMW) DNA extraction using the Qiagen MagAttract kit (Cat # 67563) and 10× Genomics Chromium library preparation. The resulting library was shipped to Admera Health for sequencing on a HiSeq X Ten using bidirectional 150-bp reads.

### African leopard sequencing

African leopard samples were obtained with permission from the Maryland Zoo (Baltimore, MD) from 1 male and 1 female leopard (Table 1). Samples consisted of previously biobanked EDTA whole blood from individuals who are now deceased (ISIS # 96040, 95007, Table 1). Whole blood was shipped on ice directly to Admera Health, where the samples were extracted using the Machery-Nagel NucleoBond HMW DNA extraction kit following the standard protocol. Samples were then shipped to the University of Georgia Genomics and Bioinformatics Core, where PacBio HiFi libraries were prepared and 2 Single Molecule, Real-Time (SMRT) cells were sequenced for each individual on the Sequel II. We note that these individuals were captured as cubs as the result of a potential poaching incident in South Africa, so the exact sample ancestry is unknown.

### Tiger genome assembly

The tiger genome was first assembled using 10×’s Supernova software (v2.1.1) using the default parameters. We output the assembly using the “–style=pseudohap,” “–minsize = 10,000,” and “–headers=sort” flags in order to output a single, pseudohaploid assembly with short headers and scaffold sizes no less than 10,000 bp. This genome was then used as the input assembly for the HiRise software from Dovetail Genomics. Briefly, the input de novo assembly and Dovetail Hi-C library reads were used as input data for HiRise, a software pipeline designed specifically for using proximity ligation data to scaffold genome assemblies (Putnam *et al.* 2016). Sequences from the Dovetail Hi-C library reads were aligned to the draft input assembly using a modified version of the SNAP read mapper (https://snap.cs.berkeley.edu). Separations of Dovetail Hi-C read pairs that were mapped to draft scaffolds were then analyzed by HiRise to produce a likelihood model for genomic distances between read pairs, and the model was then used to identify and break putative misjoins, score prospective joins, and to make joins above a defined threshold. Subsequent to scaffolding, shotgun sequences were used to close gaps between contigs. All Hi-C assembly steps were performed by Dovetail Genomics (Santa Cruz, CA, USA), and the resulting assembly returned to us.

### Snow leopard genome assembly

The snow leopard genome was first assembled using the reads generated from the 10× Genomics Chromium library with the same methods outlined in the tiger genome assembly (see *Tiger Genome Assembly*). Subsequently, Hi-C sequencing reads for the snow leopard generated by DNA Zoo (dnazoo.org) were obtained from the DNA Zoo Consortium (dnazoo.org) (Dudchenko *et al.* 2017). We used the juicer pipeline (Durand *et al.* 2016; Dudchenko *et al.* 2018) to scaffold this assembly using the Hi-C sequence data. Briefly, the input genome assembly was first indexed with BWA *index* (Li and Durbin 2009). Subsequently, generate_site_positions.py was used to generate positions of enzyme restriction sites of the input genome, with the MboI enzyme flagged as the restriction enzyme. The juicer pipeline was then run with default settings using the draft assembly and previously generated enzyme restriction file as input. Finally, the 3D-DNA pipeline was used to generate a candidate assembly by inputting the 10× draft assembly again, along with the “merged_nodups.txt” file generated in the previous step.

### Leopard genome assembly

We tested 3 assemblers (Flye; Lin *et al.* 2016; Kolmogorov *et al.* 2019, wtdbg2; Ruan and Li 2020, and Hifiasm; Cheng *et al.* 2021) for the PacBio HiFi African leopard data. Using hifiasm0.9-r289, we ran standard commands with reads from both cells for each individual as input. We used flye2.8-b1674 standard commands (reads as input) with the “–genome-size” flag set at 2.4 g. Lastly, we used wtdbg2v2.5 with reads from both cells for each individual as input, the “-g” flag set at 2.4 g and the “-x” flag set as ccs. Using the most complete assembly, as assessed by continuity statistics and gene completeness (see *Decontamination and Assembly Quality Assessment*), the primary Hifiasm assemblies were then used as input into the juicer pipeline using the same strategy as above. For this, we used Hi-C data generated by DNA Zoo (dnazoo.org) from the Amur leopard since contact mapping is unlikely to be impacted by the minimal divergence between these 2 subspecies (Corbo *et al.* 2022).

### Decontamination and assembly quality assessment

Finalized draft genomes for each species were then uploaded to NCBI for contamination screening. The contamination file generated was then used to remove possible contaminants from each assembly by masking these regions with N’s using BEDtools2.26.0 *maskfasta* (Quinlan and Hall 2010), or removed in the case of duplicated scaffolds. The cleaned genomes were then assessed for quality using Assemblathon2 (Bradnam *et al.* 2013) “assemblathon-stats” scripts. We also assessed the continuity of the genome using BUSCO (Simão *et al.* 2015) with the mammalia_odb10 and carnivora_odb10 libraries, both of which evaluate the completeness of a set of manually curated orthologous genes.

We then calculated the depth of each library. Illumina reads were mapped to each genome using BWA-MEMv0.7.17 (Li 2013) and mean depth calculated using samtools *depth* (Li *et al.* 2009) and custom scripts. Briefly, each reference genome was indexed using bwa *index* with the flags “-a bwtsw,” reads mapped using bwa *mem*, and converted to bam files using samtools view with the flags “-bS.” For the genomes generated with Pacbio HiFi data, reads were mapped to each genome using minimap2 v2.22 (Li 2018) to generate bam files and depth was calculated using the same method as for the Illumina libraries.

### Repeat masking and whole-genome alignment

The final decontaminated genome assembly for each individual was then repeat-masked using the same pipelines and settings as in Armstrong *et al.* (2020). Briefly, repeats were soft-masked first using RepeatMasker v4.0.9 based on known repeat databases from repbase using the flags “-species Carnivora,” as well as with flags “-nolow,” “-xsmall,” and “-gccalc” (Smit *et al.* 1996; Jurka *et al.* 2005). Next, the masked genome produced in the previous step was used to build a database using RepeatModeler v1.0.11 *BuildDatabase* (Flynn *et al.* 2020). We then used RepeatModeler v1.0.11 to perform de novo repeat finding on the database produced in the previous step. Finally, a masked file with both known and de novo repeats was produced by running RepeatMasker v4.0.9 with the flags “-gccalc,” and “-xsmall,” and the library produced from the previous step as input with the initial masked file. The same steps were repeated to create a “hardmask” of each genome for repeat analyses by simply replacing the “-xsmall” and “-nolow” flags with the “-a” flag. Repeats were tabulated and plotted in R (R Core Team 2014) with the ggplot2 package (Wickham 2011).

The resulting soft-masked files were then used as input to LAST v921 (Kiełbasa *et al.* 2011) for whole-genome alignment. Each genome assembly was aligned to the autosomes and the X chromosomes from the domestic cat (felCat9) following scripts from https://github.com/mcfrith/last-genome-alignments. Genome alignment was visualized using the CIRCA software (http://omgenomics.com/circa) by plotting only the alignments with a mismap score of less than 1e−9 and the longest 50% of the major alignments for each of the Felcat9 autosomes and the X chromosome. The major alignment was determined as the scaffold belonging to the query assembly that represented a majority of the alignments for each of the Felcat9 autosomes and X chromosome.

After Hi-C integration, the snow leopard assembly was found to contain a misassembly which joined cat chromosomes E1 and F1. As a result, this scaffold was manually split using juicebox tools (Durand *et al.* 2016) and subsequently checked for accuracy by producing a realignment with LAST but also through generation of an alignment dotplot with the program lastz v1.04.15 (Harris 2007). Alignments of both segments of the split scaffold and each of the E1 and F1 chromosomes were made in lastz using the flags “–notransition –step = 20 –nogapped –format=rdotplot” and resulting alignments were subsequently plotted in R (not shown). All previous stats (e.g. N50, BUSCO) were regenerated after the break was integrated.

### Clade phylogenetics and leopard ancestry verification

Using the nucleic acid sequences for the recovered single-copy BUSCOs from each subspecies, we created a phylogenetic tree using only the unique subspecies of *Panthera*, and the clouded leopard (*N. nebulosa*, dnazoo.org; *P. uncia*, this study; *P. t. altaica*, dnazoo.org; *P. t. jacksoni*, GCA_019348595.1; *P. onca*, dnazoo.org; *P. leo*, GCA_018350215.1; *P. p. pardus*, this study Ppardus1_PCG_1.0; *P. p. orientalis*, dnazoo.org). We did not include genomes that had mixed subspecies ancestry, including the “generic” tiger assembled in this paper. Each gene was aligned using MAFFT v.7.490 (Katoh and Standley 2013) using the L-INS-i algorithm. Gene alignments were trimmed using Gblocks (Castresana 2000; Talavera and Castresana 2007) v.091b. We inferred maximum likelihood individual gene trees using IQ-TREE 2 (Minh *et al.* 2020) v. 2.1.3 under default settings with 100 nonparametric bootstrap replicates and automated model selection using ModelFinder (Kalyaanamoorthy *et al.* 2017). We concatenated the maximum likelihood trees as recommended by Mirarab *et al.* (2016) and inferred the species tree using ASTRAL-III (Mirarab *et al.* 2014; Zhang *et al.* 2018) v. 5.7.8 with 100 gene-only bootstrap replicates (flag “–gene-only”) and fully annotating the inferred tree (flag “-t 2”). For comparison, we also performed the same species-tree inference analysis using the concatenated consensus maximum-likelihood gene trees. The final species trees were rooted on *N. nebulosa*.

We also concatenated all Gblocks-trimmed gene alignments into a single unpartitioned concatenated alignment. We inferred maximum-likelihood trees with 100 bootstrap models using both reversible models using automated ModelFinder model selection (model GTR + F + R8 selected) and the nonreversible 12.12 model (Woodhams *et al.* 2015). We then used FigTreev1.4.3 to visualize the tree (http://tree.bio.ed.ac.uk/software/figtree/). The final concatenated trees were rooted on *N. nebulosa*.

To more precisely examine the ancestry of the leopards sequenced here, we used principal component analysis. Bam files generated from 37 African leopards (Pečnerová *et al.* 2021) and those from the 2 putative African leopards sequenced here (see *Decontamination and assembly quality assessment*) were input into angsdv0.931 (Korneliussen *et al.* 2014) and PCAngsd (Meisner and Albrechtsen 2018) to generate a PCA. Angsd was first run using flags “-GL 2, -nThreads 64, -doGlf 2, -doMajorMinor 1, -SNP_pval 1e-6, and -doMaf 1” to generate likelihood files for input into PCAngsd. PCAngsd was then run using flags “-e 3, -minMaf 0.05” using the beagle files from the previous steps as input. PCA was visualized using R (R Core Team 2014).

## Results and discussion

### *Sequencing and* de novo *genome assemblies*

We generated sequencing for one 10× Genomics Chromium linked-read library for both a tiger and a snow leopard (Table 2). For the tiger, we generated a total of approximately 771 million paired reads (386 million read pairs), equating to an average genomic coverage of approximately 38× (Table 2). We additionally generated approximately 490 million reads from a Dovetail genomics Hi-C chromatin library, for an average genomic coverage of 29× (Table 2). For the snow leopard, we generated approximately 856 million reads for an average coverage of 40× (Table 2).

**Table 2.** Number of reads generated and average depth of sequencing for the tiger and the snow leopard.

	GenTig	Puncia
Number of 10× chromium linked reads	771,299,128	856,081,223
Average depth (10×)	37.85×	40.04×
Number of Hi-C reads	490,780,448	NA
Average depth (Hi-C)	28.74×	NA

Initial 10× assemblies for the tiger and the snow leopard generated 1,343 and 2,838 scaffolds, respectively. Both assemblies had similar contig N50 values (∼320 kb; Table 3); however, the 10× assembly for the tiger had almost double the scaffold N50 and half of the scaffold L90 of the snow leopard (Table 3). Interestingly, this does not appear to be a factor of the average input molecule size, which was 36,584 bases for the tiger and 36,914 bases for the snow leopard. However, the tiger did have a lower % of duplicate sequences (8.73% in the tiger compared to 14.86% in the snow leopard), and also revealed a difference in the number of molecules extending 10 kb on both sides (111 in the tiger and 80 in the snow leopard). The snow leopard sample is additionally less heterozygous than the tiger, and the Supernova assembler reported a distance of 5.1 kb between heterozygous SNPs in the snow leopard, compared to only 1.17 kb in the tiger. While this would theoretically affect the assembler’s ability to phase the assemblies, it is not clear whether it would impact assembly quality. Indeed, the difference in coverage or molecule size alone does not appear to explain the differences between the assembly quality of the tiger and the snow leopard, since a previous study showed that 10× Genomics linked-read data is robust across a range of coverages over 25× and is only greatly impacted by the mean molecule size (Armstrong *et al.* 2019).

**Table 3.** Statistics of 10× Genomics Chromium assemblies for tiger and snow leopard.

	Puncia (10× only)	GenTig (10× only)
Total length	2,434,288,486	2,407,822,281
Scaffolds	2,838	1,343
Contigs	16,324	15,398
Contig N50	322,039	328,032
Scaffold N50	27,928,322	44,539,166
Scaffold L90	96	66

Using PacBio HiFi sequencing, we generated data from 2 SMRT cells each for 2 putative African leopards. Of these, between ∼32% and 43% of the reads converted to circular consensus sequences (CCS), which translated to a final approximate depth of 25× and 27× for Ppardus1 and Ppardus2, respectively (CCS; Table 4). The low number of reads passing filter is likely due to the age and degradation of the samples, which were taken from each leopard postmortem and stored in the Maryland Zoo biobank. Ppardus1 died in 2016 and Ppardus2 died in 2013.

**Table 4.** Number of reads generated and average depth of sequencing for each African leopard.

	Ppardus1 cell 1	Ppardus1 cell 2	Ppardus2 cell 1	Ppardus2 cell 2
ZMWs generating circular consensus sequences	1,908,091	1,695,169	1,893,243	2,001,644
ZMWs filtered	3,424,371	3,674,675	2,555,091	3,013,882
Average depth	25.06×		26.98×	

ZMW, zero-mode waveguides generated from SMRT sequencing.

Despite sample degradation and the resulting decrease in average coverage, data for both leopards were successfully used as input in 3 different genome assembly programs. In general, all assemblers produced draft assemblies of approximately 2.3–2.4 Gb, but Hifiasm (Cheng *et al.* 2021) produced the assemblies with the fewest contigs (Ppardus1, 429; Ppardus2, 420; Table 5), and smallest contig L90 (Ppardus1, 108; Ppardus2, 94; Table 5). Comparatively, wtdbg2 (Ruan and Li 2020) generated a draft assembly with 647 and 532 contigs for Ppardus1 and Ppardus2, respectively, while Flye (Kolmogorov *et al.* 2019), produced the largest number of draft scaffolds and the smallest contig N50s (Table 5). However, all long-read assemblers produced substantially more contiguous genomes than those produced by 10× Genomics linked-read technology, reiterating the utility of long-reads in genome assembly.

**Table 5.** Statistics comparing various pacbio assembly tools for Ppardus1 and Ppardus2.

	*Hifiasm (Ppardus1)*	*Hifiasm (Ppardus2)*	wtdbg2 (Ppardus1)	wtdbg2(Ppardus2)	Flye (Ppardus1)	Flye (Ppardus2)
Total length	2,441,163,814	2,436,970,322	2,377,391,774	2,377,871,889	2,416,305,443	2,417,534,344
Contigs	429	420	647	532	2,012	1,942
Contig N50	29,007,632	33,312,980	20,611,268	25,081,460	8,969,496	12,809,951
Contig L90	108	94	122	106	335	225

Assemblies chosen for Hi-C scaffolding are italicized.

### *Hi-C integration and comparison with other* Panthera *genomes*

We lastly integrated Hi-C data for all 4 draft *de novo* assemblies produced in this study. In all cases, integrating Hi-C data led to an increase in continuity and a substantial decrease in the scaffold L90 (Table 6). All assemblies except for the snow leopard have more than 90% of their genome contained in 19 or fewer scaffolds (the felid karyotype is 2*n* = 38), reinforcing that these are all highly continuous, chromosome-scale assemblies.

**Table 6.** Assembly statistics of final assemblies for all species and individuals.

	Ppardus1_PCG_1.0 (Pacbio + Hi-C)	Ppardus2_PCG_1.0 (Pacbio + Hi-C)	Puncia_PCG_1.0 (10× + Hi-C)	GenTig_PCG_1.0 (10× + Hi-C)
Total length	2,441,326,314	2,437,106,822	2,434,048,986	2,407,461,690
Scaffolds	381	389	2,642	1,202
Contigs	706	674	16,601	15,399
Contig N50	22,977,662	25,050,590	321,033	327,402
Scaffold N50	126,810,544	158,060,271	112,063,229	145,225,440
Scaffold L90	19	15	20	16

Compared to several of the previous tiger assembles that are publicly available (PanTig1.0, Cho *et al.* 2013; PanTig1.0-HiC, dnazoo.org; Maltig1.0, Armstrong *et al.* 2019), GenTig_PCG_1.0 improved the contig N50 by almost 4-fold compared to PanTig1.0 and generated a comparable scaffold N50 to the improved PanTig1.0-HiC from DNAZoo and the ptimat1.1 assembly (Tables 6 and 7). However, the ptimat1.1 assembly, which was generated using long-read Pacbio sequencing and a trio-binning approach (Bredemeyer *et al.* 2021), has a larger contig N50 compared to all other assemblies. The newly generated snow leopard assembly (Puncia_PCG_1.0) showed a 2-fold increase in the contig N50 (159.87–321.03 kb; Tables 6 and 7) and showed a similar scaffold N50 compared to the DNAZoo snow leopard assembly (PanUnc-HiC). The 2 African leopard assemblies showed an over 500-fold increase in the contig N50 compared to the published Amur leopard assemblies (PanPar1.0 and PanPar1.0_HiC; Tables 6 and 7). The African leopard genomes, which were assembled using PacBio HiFi data, also had higher contig N50s compared to the assemblies generated by 10× Genomics Chromium linked-read sequencing (Tables 6 and 7). Interestingly, the 2 leopard assemblies had slightly different contig N50 values, which is likely explained by the number of reads that were converted to CCS (Table 4). Overall, long-read sequencing consistently improves overall assembly continuity, especially impacting the distribution of contig lengths in the assembly.

**Table 7.** Summary statistics for other publicly available assemblies for the tiger, snow leopard, and leopard.

	PanUnc-HiC (DNAZoo)	PanPar1.0 (GCA_001857705.1)	PanPar1.0-HiC (DNAZoo)	PanTig1.0 (GCA_000464555.1)	PanTig1.0-HiC* (DNAZoo)	Maltig1.0* (GCA_019348595.1)	ptimat1.1 (GCA_018350195.2)
Total length	2,408,534,935	2,578,019,207	2,578,248,701	2,391,383,019	2,391,065,193	2,424,644,036	2,408,695,688
Scaffolds	109,240	50,377	50,025	1,055	1,478	10,077	75
Contigs	159,866	162,036	162,190	126,631	126,369	36,281	140
Contig N50	65,012	40,598	40,542	39,172	39,142	185,547	74,391,967
Scaffold N50	141,103,905	21,701,857	155,751,443	8,860,407	145,958,240	21,297,815	146,942,463
Scaffold L90	17	135	16	15	284	109	16

Assemblies marked with * were used in phylogenetics analysis.

**Fig. 1.**
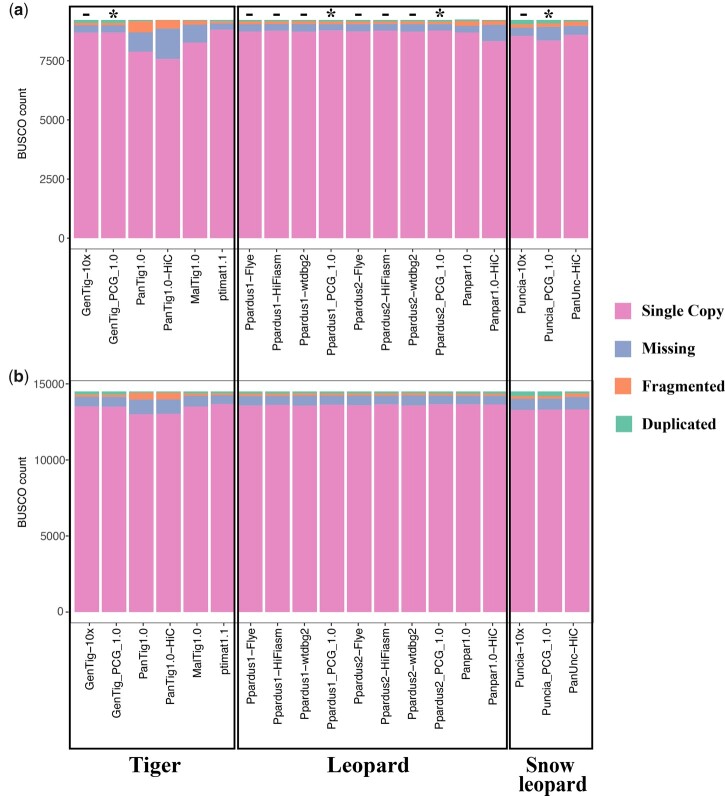
BUSCO scores from the (a) mammalia_odb10 and (b) carnivora_odb10 datasets across all draft (−), final (*), and previously published (unmarked) assemblies.

To further evaluate the quality of the assemblies, we ran BUSCO (Simão *et al.* 2015) on all available genomes for the leopard, tiger, and snow leopard using both the mammalia_odb10 and carnivora_odb10 datasets. For the most part, all assemblies generated with linked-read or long-read technology had minimal differences in BUSCO statistics (Fig. 1). Assemblies generated from both African leopards had a higher number of single-copy BUSCOs compared to either of the assemblies generated with the linked-read technology, except those generated with the wtdbg2 (Redbean; Ruan and Li 2020) assembler (Fig. 1). Overall, there were slight improvements in the number of complete and single-copy genes when the assemblies were upgraded with Hi-C, but this effect was minimal and only involved a few genes (Fig. 1). Interestingly, all snow leopard genomes (even those upgraded with Hi-C or built with linked-read technology) showed a large number of missing genes relative to other species, suggesting that there may have been loss of conserved genes in that lineage during its evolutionary history. The pattern is surprising, given that improvements in contig N50 scores across other genomes corresponded to a decrease in the number of missing BUSCOs. These results were more obvious when using the mammalia_odb10 dataset (*N* = 9,226 genes; Fig. 1a) compared to the carnivora_odb10 dataset (*N* = 14,502 genes; Fig. 1b). Whether this signal represents a true decrease in the number of genes in the snow leopard relative to other carnivores or not requires further investigation.

### Repeat content

**Fig. 2.**
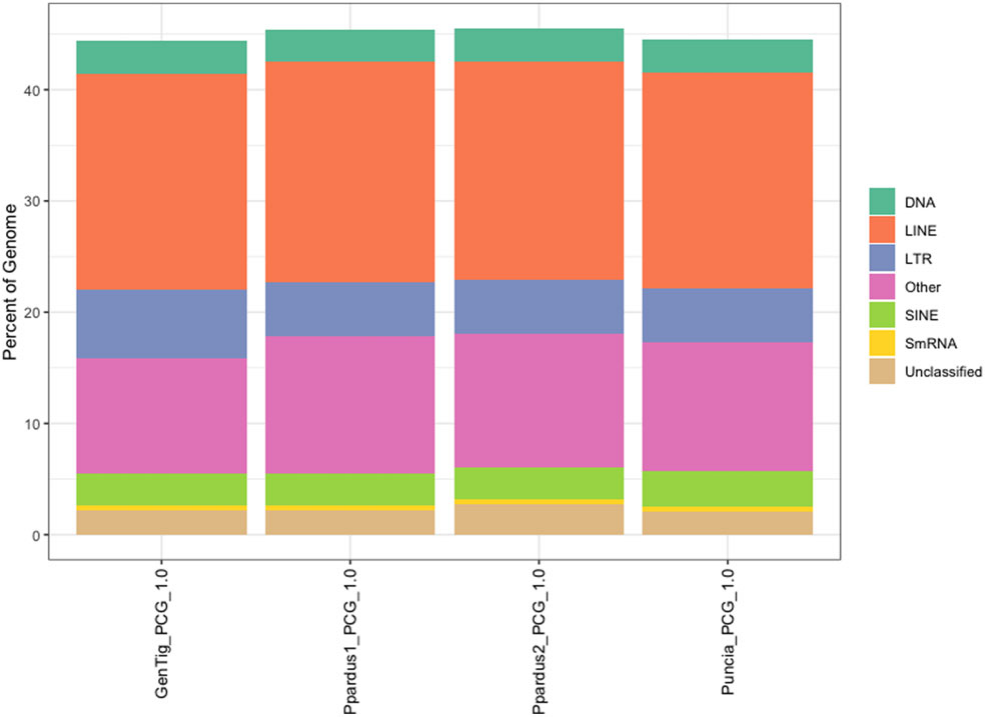
Percentages of various repetitive elements classes for de novo *Panthera* genome assemblies.

We evaluated the repetitive content of each new assembly using a combination of homology and *de novo* repeat finding tools. Repeat content was similar across the assemblies (Fig. 2, 44.35–45.51%), which is consistent with previous repeat content estimates for *Panthera* (Armstrong *et al.* 2020). However, we did find that both of the long-read leopard assemblies contained on average higher repetitive content compared to all other assemblies. Although we make no direct comparison here to a short read assembly from the same individuals, it does suggest that repeat discovery is improved by long-read sequencing.

### Whole-genome alignment

**Fig. 3.**
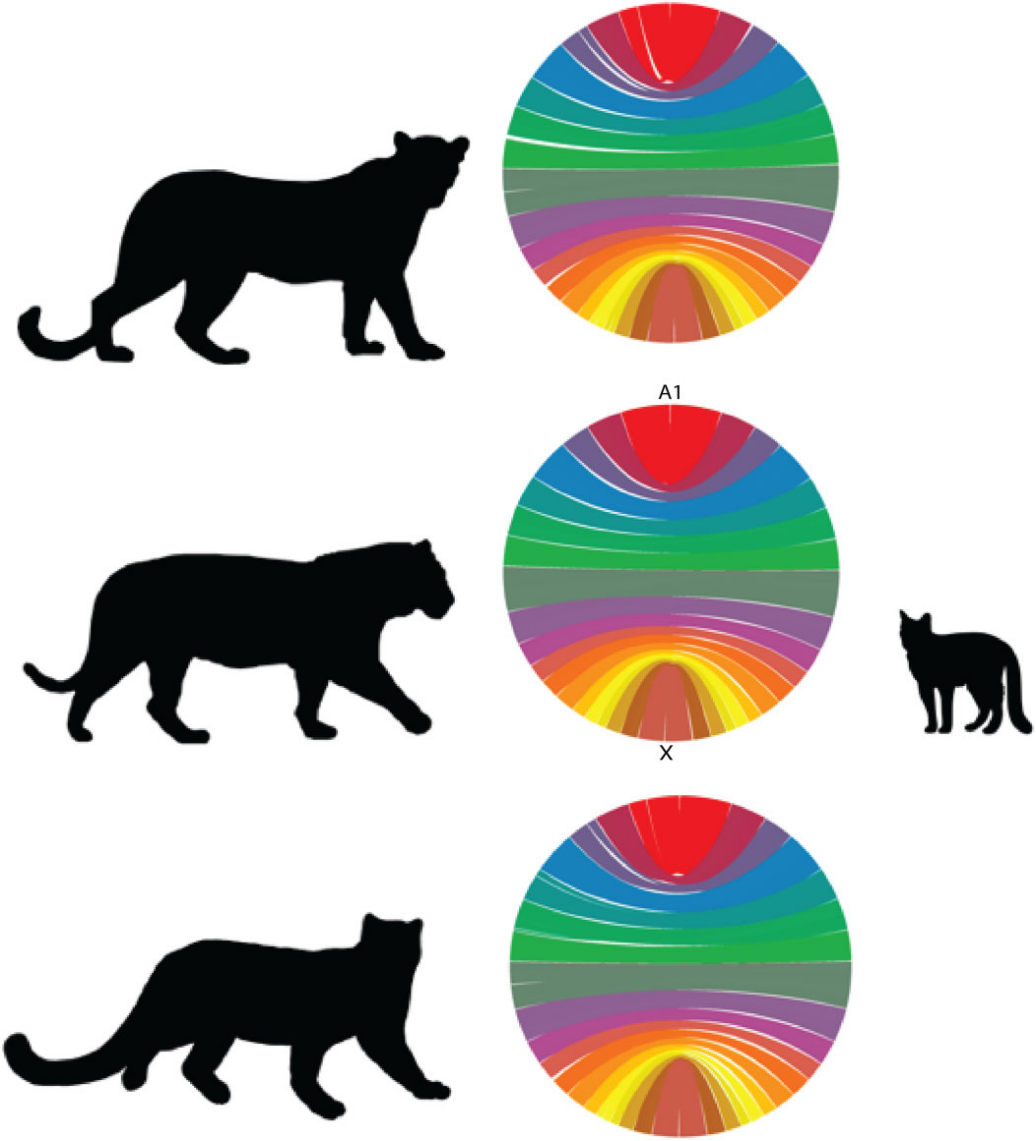
Whole-genome alignments for (from top to bottom) leopard, tiger, and snow leopard (left) to the domestic cat (right). Silhouettes from phylopic.org: Snow leopard by Gabriela Palomo-Munoz; leopard, no license; tiger by Sarah Werning; domestic cat, no license. http://creativecommons.org/licenses/by/3.0/.

We generated whole-genome alignments for the domestic cat and the final *de novo* genomes for the leopard, snow leopard, and tiger. The whole-genome alignments reiterate the conserved karyotype of the *Panthera* clade and additionally show the high continuity of each assembly (Fig. 3). In addition, we were able to assemble a majority of the X chromosome in each species. In the domestic cat, the X chromosome is estimated to be approximately 131.8 Mb (GCA_000181335.5). In Ppardus1_PCG_1.0, we identified a scaffold corresponding to the X chromosome that was 126.8 Mb in size, a corresponding scaffold of 125.2 Mb in Ppardus2_PCG_1.0, a 120.3 Mb scaffold in GenTig_PCG_1.0, and lastly, for Puncia_PCG_1.0, a scaffold of 122.7 Mb. These improved assemblies will enable further investigation into the peculiar lack of divergence of the X chromosome in the *Panthera* lineage, as reported by Li *et al.* (2016).

### Clade phylogenetics and leopard ancestry verification

**Fig. 4.**
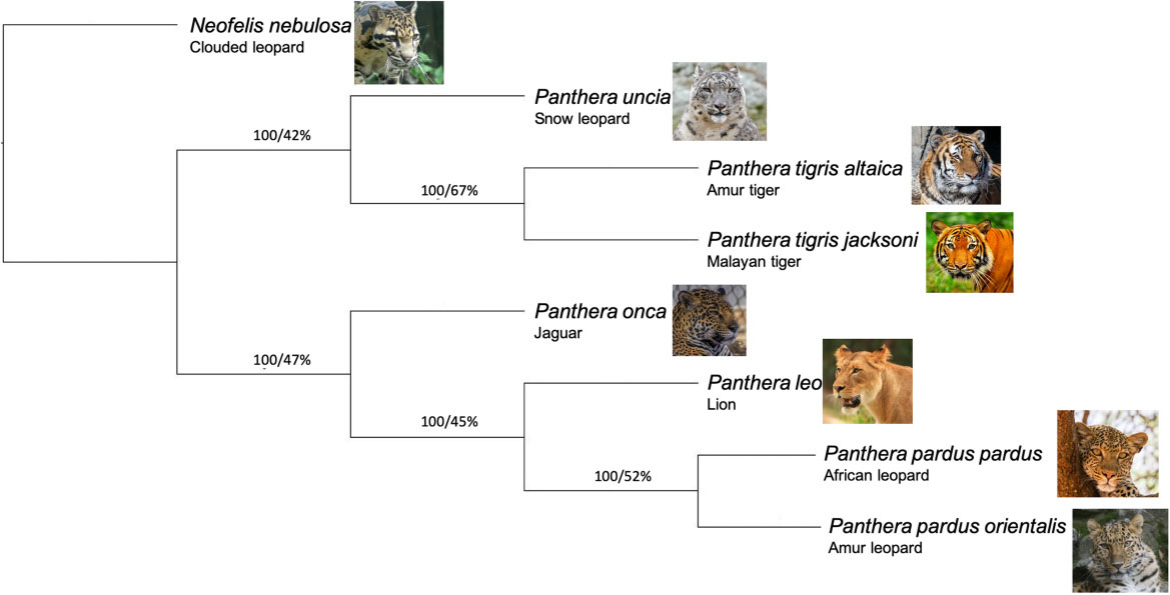
Cladogram representing phylogenetic results from ASTRAL-III using 8,719 BUSCO genes for the *Panthera* clade and an outgroup, the clouded leopard (*Neofelis nebulosa*). Plotted from results of the ASTRAL-III species tree using the best individual gene trees. Notation indicates bootstrap/quartet score for each node.

Using highly conserved genes from BUSCO, we built a tree with 1 sample of each (sub)species of the *Panthera* clade with the available genomes, using the clouded leopard (*N. nebulosa*) as the outgroup (Fig. 4). The final tree was built using 8,719 single-copy orthologs, the largest orthologous gene set ever analyzed for this clade and the first to include multiple subspecies from several members of *Panthera*. The overall species tree topology is supported with high bootstrap support (100). This topology is not completely unexpected because only conserved coding regions were included. Our results agree with previous studies that find the most discordance in the lion-leopard-jaguar trio, where previous studies have suggested a possible species tree topology placing the lion sister to the jaguar, rather than the leopard (Li *et al.* 2016, 2019). These high-quality genomes can be used to further investigate the phylogenetic discordance across the clade using large numbers of orthologous gene sequences, as used here, or genome alignments that are not reliant on mapping to divergent species, as in previous studies (Li *et al.* 2016, 2019).

**Fig. 5.**
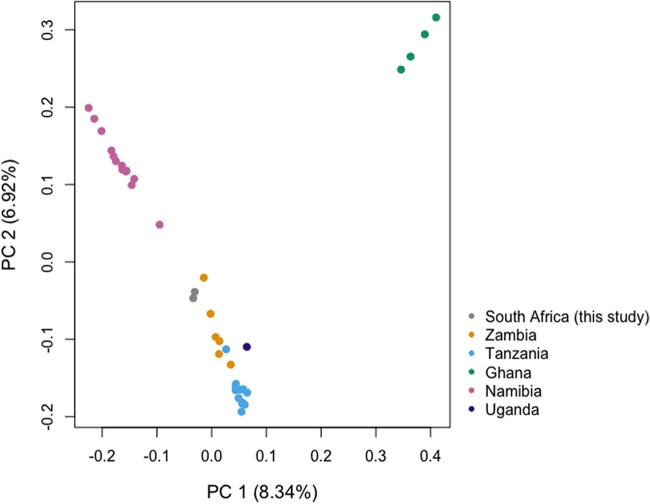
Principal component analysis showing PC1 vs PC2 of putative African leopards from this study, and previously published whole-genome data from African leopards. Samples are colored by source locations identified in Pečnerová *et al.* (2021).

Using principal component analysis, we then analyzed 37 previously sequenced African leopards of known provenance from Pečnerová *et al.* (2021) and the 2 leopards sequenced in this study (of unknown provenance) with putative African ancestry. We found that these 2 leopards fall well-within the bounds of what would be expected from an African leopard (Fig. 5). An additional study analyzing multiple leopard subspecies (Paijmans *et al.* 2021) showed that African leopards are highly differentiated from every other leopard subspecies across their genomes, implying that we would expect that the leopards examined here would not cluster with the other African leopards included here if they were not of African ancestry. The two leopards group most closely to leopards from Zambia, but additional data will be needed from across the African leopard range to identify the specific ancestry of these individuals. Although some additional African leopards were sequenced as a part of Paijmans *et al.* (2021), most of these are from historic specimens. With continued land use change and habitat loss, a comprehensive modern examination of leopard structure across their range would assist in the identification of habitat corridors and further aid in confirming the source identification of poached individuals and other trafficked materials, such as the leopards here.

## Summary assessment of genome assembly and annotation

Using various technologies, we constructed several new chromosome-level assemblies for the tiger, the snow leopard, and the African leopard. We also use biobanked samples for PacBio sequencing and successfully built 2 high-quality assemblies from these samples. Our analysis showcases that older samples, subject to proper storage, can successfully be used for HMW DNA extraction and sequencing, reinforcing the utility and importance of biobanking initiatives. Although several genomes exist for the tiger, we added a novel genome assembly for a captive tiger of unknown ancestry, an improved assembly for the snow leopard, and the first assemblies for the African leopard. We show that in all cases, incorporation of Hi-C data substantially increases the continuity of the genomes to approximately chromosome level. In addition, we find that as in previous studies, the *Panthera* clade is remarkably conserved at the karyotypic level, and according to strict filtering parameters find no apparent large chromosome-level inversions between the domestic cat and any of the genomes examined. These high-quality reference genomes will help to reveal the evolutionary history of this important clade and are highly relevant to the conservation of *Panthera*.

## Data Availability

All sequence data and final assembly files for the snow leopard have been deposited under NCBI BioProject PRJNA602938. All sequence data and final assembly files for the tiger have been deposited under NCBI BioProject PRJNA770127. All sequence data and final assembly files for the leopard have been deposited under NCBI BioProject PRJNA781109.
